# Predictive value of bile acids as metabolite biomarkers for gallstone disease: A systematic review and meta-analysis

**DOI:** 10.1371/journal.pone.0305170

**Published:** 2024-07-25

**Authors:** Xu Han, Juan Wang, Yingnan Wu, Hao Gu, Ning Zhao, Xing Liao, Miao Jiang

**Affiliations:** 1 Institute of Basic Research in Clinical Medicine, China Academy of Chinese Medical Sciences, Beijing, China; 2 Department of Traditional Chinese Medicine, Inner Mongolia People’s Hospital, Hohhot, China; Sapporo Medical University, JAPAN

## Abstract

**Background:**

The profiles of bile acids (BAs) in patients with gallstone disease (GSD) have been found to be altered markedly though in an inconsistent pattern. This study aims to characterize the variation of the BA profiles in GSD patients, thereby to discover the potential metabolite biomarkers for earlier detection of GSD.

**Methods:**

Literature search of eight electronic database in both English and Chinese was completed on May 11, 2023. The qualitative and quantitative reviews were performed to summarize the changes of BA profiles in GSD patients compared with healthy subjects. The concentrations of BAs were adopted as the primary outcomes and the weighted mean differences (WMDs) and 95% confidence interval (CI) were generated by random-effects meta-analysis models.

**Results:**

A total of 30 studies were enrolled which included 2313 participants and reported the 39 BAs or their ratios. Qualitative review demonstrated serum Taurocholic Acid (TCA), Glycochenodeoxycholic acid (GCDCA), Glycocholic acid (GCA), Taurochenodeoxycholic acid (TCDCA), Glycodeoxycholic acid (GDCA) and Deoxycholic acid (DCA) were significantly increased in GSD patients compared with healthy subjects. Meta analysis was performed in 16 studies and showed that serum Total BAs (TBA) (WMD = 1.36μmol/L, 95%CI = 0.33; 2.4) was elevated however bile TBA (WMD = -36.96mmol/L, 95%CI = -52.32; -21.6) was declined in GSD patients. GCA (WMD = 0.83μmol/L, 95%CI = 0.06; 1.6) and TCA (WMD = 0.51μmol/L; 95%CI = 0.18; 0.85) were both increased in serum sample; TCDCA (WMD = 2.64mmol/L, 95%CI = 0.16; 5.12) was rising, however GCDCA (WMD = -13.82mmol/L, 95%CI = -21.86; -5.78) was falling in bile sample of GSD patients. The level of serum DCA in the GSD patients was found to be increased by using chromatography, yet decreased by chromatography mass spectrometry.

**Conclusion:**

The profiles of BAs demonstrated distinctive changes in GSD patients compared with healthy control subjects. Serum GCA, TCA and GCDCA, as the typically variant BAs, presented as a potential marker for earlier diagnosis of GSD, which could facilitate early prophylactic intervention. Yet, further validation of these biomarkers by longitudinal studies is still warranted in the future.

**PROSPERO** registration number CRD42022339649.

## Introduction

Gallstone disease (GSD) is one of the most common gastrointestinal diseases with a high prevalence rate reaching 20% in developed countries and 10% in China [1, 2]. GSD can be classified as cholesterol gallstone, pigment gallstone (black and brown), and mixed gallstone according to the calculus composition and appearance [3]; among which cholesterol cholelithiasis is the most common type (70%), forming cholesterol crystals [4]. Due to the lifestyle change and economic development, the prevalence rate of GSD keeps increasing worldwide year by year, consequently the risks of the severe complications such as cholecystitis and pancreatitis rise significantly [5, 6]. In addition, as the most important risk factor for gallbladder cancer [7], GSD causes a huge economic burden worldwide, for example, the total expenditure of biliary tract diseases in the United States has reached 16.9 billion dollars in 2021 [8].

The typical symptoms of GSD include intense abdominal pain, fever, nausea, vomiting and jaundice [3]. Current mainstream therapies consist of pain relief with analgesics, oral litholysis with ursodeoxycholic acid (UDCA), and routine open or laparoscopic cholecystectomy [9], with the therapeutic goals aiming at controlling symptoms, avoiding recurrence, and preventing complications. Once lithogenesis occurs and the gallstone reaches a certain size, cholecystectomy is the gold standard therapy for symptomatic GSD patients with biliary pain or complications [10, 11]. However, nonspecific postsurgical gastrointestinal symptoms like persistent abdominal pain and dyspepsia occur in up to 10% of cases [12]. Moreover, multifarious post operative complications, for example injuries of bile ducts, bile leaks, bleeding, intestinal injuries, and infection, may occur. These will cause the multiplication of patient pain and the significant rise in healthcare costs. Conservative treatment is also popular in clinic, for example, UDCA is prescribed to dissolve the stone, yet the indications are limited, and the risk of gallstone recurrence is as high as 30–43% within 3 to 5 years [13, 14]. Thus, an optimal strategy for GSD management is the primary prevention of the gallstone formation in advance, rather than anti-symptomatic treatment after diagnosis.

In order to accomplish the prophylactic treatment of gallstones, one of the imperative steps is to discover the early warning indicators of gallstone occurrence before lithogenesis, so as to make an earlier diagnose. Bile acids (BAs) are regarded as the best potential candidates given their physiological functions and chemical properties.

BAs are synthesized in hepatocytes and secreted into the intestinal tract, as an important component of bile stored in the gallbladder [15]. The homeostasis of BAs plays a key role in the prevention of gallstone formation, and a slight transformation of BAs can trigger the drastic effect of gallstone development. Meanwhile, BAs also play a substantial role in digestion, absorption, and metabolism. As a signaling molecule, BAs regulate various receptors like Farnesoid X receptor (FXR) and G-protein coupled receptor (GPCR). Therefore, BAs metabolism is also related to glucose homeostasis, lipid and lipoprotein metabolism, energy expenditure, intestinal motility, bacterial growth, inflammation, and the liver-gut axis [16, 17], which are all involved in the lithogenesis. Therefore, an increasing number of researches have focused on the underlying value of BAs in the prediction, diagnosis, and treatment of various diseases, especially hepatic and gall diseases.

Additionally, as a group of substances, the changes of BA profiles are complex, the current metabonomic technologies can provide a possibility to determine the overall changes of BA profiles qualitatively and quantitatively, thus to characterize the real-time state of the body with BA profiles.

Up to now many studies have demonstrated that the levels of some BAs are significantly associated with the development of GSD [18–20]. However, due to the diversity of the metabonomic technologies and biological samples, there is still lack of a consistent and comprehensive conclusion on the specific changes of BA profiles in GSD patients. Thus, a systematic review is necessitated based on the published literature. Our study aims to provide a comprehensive summary about BA profiles in GSD and analyzes the difference in diverse samples compared to healthy subjects, thus to determine the characteristic BAs as the potential metabolite biomarkers for predicting GSD.

## Methods

Our systematic review was completed under the guidance of the PRISMA 2020 statement [21]. The protocol was registered in the International Prospective Register of Systematic Reviews (PROSPERO) database (No. CRD42022339649).

### Search strategy

Electronic database searches were performed in four English databases including PubMed, the Cochrane Library, EMBASE and Web of Science, and four Chinese databases including China Biology Medicine Disc, China National Knowledge Infrastructure, Wanfang databases and VIP Information Resource Integration Service Platform with English and Chinese, respectively. The search strategy combined the Medical Subject Heading terms, key words, and word variants for “Gallstones” and “Metabolomics” (S1 Table). No time restriction was applied. Human studies were limited in order to get more accurate search results.

### Study selection and eligibility criteria

All retrieved records were imported into Endnote X9, and duplicated records were deleted. Two authors (JW and YNW) independently screened for eligibility of all papers. All records were screened based on title and abstract firstly, then the full-texts of the studies which met our inclusion criteria were obtained for further screening. Any discrepancies and divergences were discussed and consensus was reached under the help of the third researcher (MJ). A Senior Researcher (XL) guided the entire process.

A study would be included when meeting all of the following inclusion criteria: 1) clinical study focusing on adult GSD patients; 2) including at least one control group of individuals without GSD; 3) using metabolomic technology to analyze the biological samples (blood, urine, or feces) for determining the BA profiles; 4) the variation trend or concentration of the BAs being reported. Studies that contained patients with various acute cholecystitis, gallbladder perforation complicated with diffuse peritonitis, or patients who were receiving BAs drugs like UDCA were excluded. If multiple papers were published on the same cohort, the study with the most complete information was included to avoid population overlap. Studies without available full texts or with insufficient information were removed.

### Risk of bias assessment

The risk of bias and methodological quality of the included studies were measured by using the Newcastle-Ottawa Scale (NOS) (http://www.ohri.ca/programs/clinical_epidemiology/oxford.asp) by two independent researchers (JW and YNW), as all included studies were case-control studies. NOS is a tool developed to assess the quality of observational study by a “star system” with a maximum achievable score of 9 stars. The studies were assessed based on included selection, comparability and exposure [22]. We considered studies which obtained equal to or over 7 stars as high quality.

### Data collection

Two researchers (HG and NZ) applied a unified and standardized approach to extract the following data: the name of the first author; year of publication; population location; language; type of study design; the sample size; age of each group; type or location of gallstones (cholesterol gallstone, pigment gallstone, mixed gallstone or cholangiolithiasis); results of the clinical examination such as body mass index (BMI), levels of triglyceride (TG) and cholesterol (if available); biological sample (blood, urine, feces or bile) and patient status at the time of sample collection (fasting, postprandial or intraoperative); metabolomics technique such as gas chromatography-mass spectrometry (GC-MS), liquid chromatography-mass spectrometry (LC-MS) or nuclear magnetic resonance (NMR); method of metabolites identification; the variation trend and concentration of the BAs in both GSD population and control group; validation study in the independent cohort (if available). Corresponding authors were contacted for raw data when they were not available from published documents. If their authors can’t be contacted, Web Plot Digitizer (V.4.2, San Francisco, California: Ankit Rohatgi, 2019) was used to extract data from graphs in article. All acquired data were stored and managed by using Microsoft Excel files.

Metabolites were identified according to their provided names or database ID. To avoid simultaneous use of synonymous names of different metabolites, we have annotated names and IDs from the Human Metabolome Database (https://hmdb.ca/metabolites) and Chemical Entities of Biological Interest (https://www.ebi.ac.uk/chebi/).

The concentration units were uniformly converted into μmol/L for blood samples and mmol/L for bile samples with the molecular weight (g/mol) which obtained from PubChem (https://pubchem.ncbi.nlm.nih.gov/) and showed in S2 Table. The median with quartile or interquartile range was converted into mean with standard deviation (SD) by using the statistical method and the formula, respectively. The statistical method combined skewness and a new piecewise function based on the size of sample [23–25]. The formula was provided by the Cochrane handbook for Systematic Reviews of Interventions and shown in S3 Table.

### Data synthesis

The qualitative review was performed to summarize the changes of BA profiles between GSD and control group by counting the frequency of different species of BAs in identified studies.

Then, the quantitative review was performed by meta-analyses with the concentrations of BAs as the primary outcomes. Notably, we only meta-analyzed the estimates of metabolites that were reported in at least two different studies. The random-effects model was selected because it incorporates both within- and between-study components of variance. The data were expressed as weighted mean difference (WMD) with 95% confidence interval (CI) values for continuous outcomes. I^2^ statistic measured heterogeneity, and over 30% of I^2^ was considered as substantial heterogeneity (http://handbook.cochrane.org). Sensitivity analyses were performed by using sequential omission of individual studies and removing the high-risk studies. Subgroup analysis was performed based on analytic technique. Funnel plots and the Egger’s test was applied to assess publication bias when feasible (10 or more studies) [26, 27]. All the data synthesis were performed by using R software (Version 3.6.2) with meta package.

## Results

### Study characteristics

**Fig 1:**
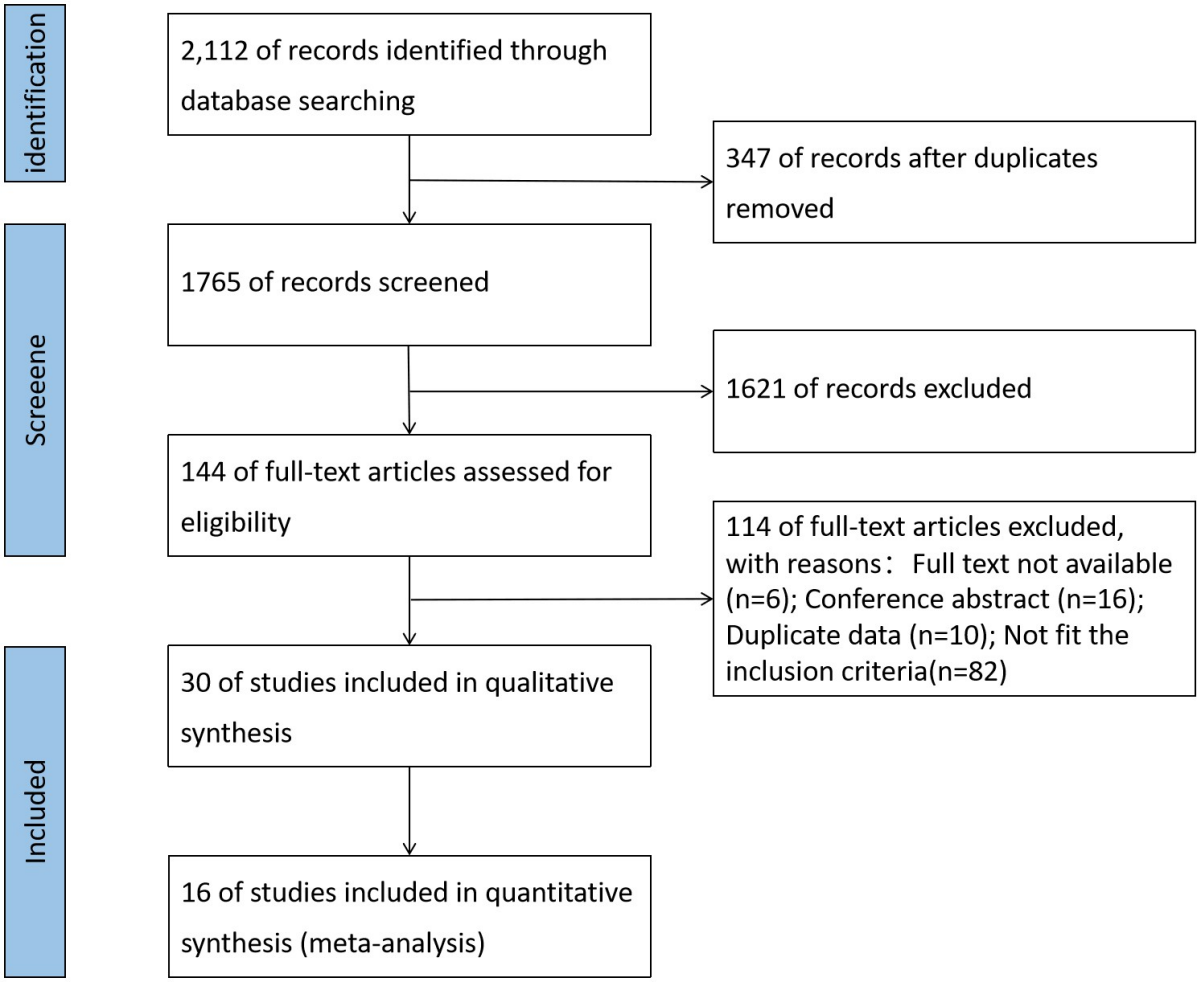
Flow chart of study selection.

The literature searching was completed on May 11, 2023. The total 2112 of records were identified. After removing duplications, there remained 1765 studies; then 1621 studies were excluded after title and abstract screening. Among the 144 records getting into the full-text review, 114 were excluded: 82 records for not compliance with the inclusion criteria, 16 for being conference abstract, 6 for unavailability and 10 for duplication. 30 studies [18, 19, 28–55] were finally included with a pooled sample size of 2313 participants (1391 cases in the case group and 922 in the control group). The flow chart is shown in the Fig 1.

All the included studies were case-control studies, and were published between 1973 and 2022. These studies were conducted in China [18, 19, 28, 35, 38–55], America [36], Argentina [34], Canada [37], Czech Republic [29], England [33], Israel [31], Italy [32] and Spain [30]. All patients in the case group were diagnosed with cholelithiasis, therein 19 studies [18, 30–34, 37–39, 43–50, 52, 55] were focusing on cholecystolithiasis; 5 studies [28, 29, 38, 46, 49] on choledocholithiasis; 6 studies [19, 35, 36, 51, 53, 54] on cholesterol gallstones. In 2 studies [28, 37] the patients were diagnosed with GSD complicated with chronic cholecystitis; in another study [19], the patients with cholesterol gallstone were pooled with cholesterol polyp together based on the clinical pathological results.

The subjects in the control group included four types of populations: healthy controls; liver donors without GSD; individuals without GSD (not declaring whether they had any disease or not); and non-GSD patients but with diseases including but not limited to abdominal injury [44], gastroduodenal ulcer and early gastric cancer [47], hepatic tumor [19], hepatic cyst and hepatic hemangioma [39], upper gastrointestinal disease [37, 48], brain trauma [54], brain accident or stroke [30] and other diseases that required upper abdominal surgery [53].

In these studies, BA profiles in the biological samples (blood, bile, or feces) were determined by using chromatogram, chromatography mass spectrometry or NMR. BAs were identified by internal standards in 26 included studies [18, 19, 28, 29, 32, 33, 35–42, 44–55] and by spectral library search in 3 studies [28, 30, 42]. The spectra were reported in 7 articles [39, 43–46, 51, 53], and RT and m/z of metabolites were reported in 2 articles [41, 46]. The general characteristics of included studies are presented in Table 1.

**Table 1:** The general characteristics and NOS scores of included studies.

Reference	Population location	Language	Sample size of case group (n = M/F)	Age of case group (x±s,or median with range)	Sample size of control group (n = M/F)	Age of control group (x±s,or median with range)	Technique	Biological samples and stata of samples collection	NOS
2022 Suli Jin	China	Chinese	48 = (16,32) and 11 = (2,9) ^e^	54.08±12.68 and 58.36±14.61 ^e^	26 = (10,16)	48.35 ±11.33	UPLC-MS/MS	Serum (fasting)	7
2022 Man Yang	China	Chinese	23 = (9,14)	14 (< = 55); 9 (>55)	21 = (12,9)	21 < = 55	UPLC-MS	Plasma (fasting)	5
2020 Linshi Wu	China	English	292 = (107,185)	51.9±13.41	13 = (6,7)	51.9±13.41	LC-MS	Plasma (fasting)	6
2020 Yinhuan Duan	China	Chinese	33 = (15,18)	52.5±18.1	51 = (16,35) *	40.6±13.3	UPLC-MS/MS	Serum (fasting)	6
2020 Zhibo Wang	China	Chinese	80 = (40,40)	56.8±4.8	80 = (40,40)	53.9±5.5	HPLC-MS	Serum (fasting)	6
2018 Zexu Chen	China	Chinese	30 = (14,16)	50.32±6.16	30 = (16,14)	48.73±5.25	UPLC-Q-TOF-MS	Serum (fasting)	8
2014 DongQing Ge	China	Chinese	19 = (6,14)	54.9(24–73)	10 = (4,6)	57.7(40–76)	HPLC/MS/MS	Serum (fasting)	5
2001 Dayi Chen	China	Chinese	62 = (21,41) and 24 = (11,13) ^e^	48.5 and 47.5 ^e^	39 = (16,23) *	45	RP-HPLC	Serum (fasting)	6
2000 Linzi A. Thomas	England	English	20	NG	20	NG	GC-MS	Serum (fasting)	6
1999 Chunhua Zong	China	Chinese	20 = (7,13)	57.90±12.8	20 = (11,9) *	37.53±9.8	RP-HPLC	Serum (fasting)	5
1998 Han Tian-Quan	China	English	151 = (63,88)	52.6±11.5	256 = (169,87) *	50.0±10.3	GC ^c^	Serum (fasting)	5
1995 Xueping Ma	China	Chinese	82	NG	30 *	NG	HPLC ^d^	Serum (fasting)	5
2021 Yuan Liao	China	Chinese	31 = (9,22)	51±15	9 = (2,7)	46±10	UPLC-ESI-MS/MS	Bile (during operation)	6
2020 Jingli Cai ^a^	China	English	35 = (15,20)	45.23±2.34	20 = (11,9)	49.45±1.65	LC-MS	Bile	6
2019 Stanislav Rejchrt	Czech Republic	English	25	NG	20 *	NG	UPLC-MS	Bile (during operation)	4
2019 Natalia Molinero	Spain	English	14 = (3,11)	52±14.2	13 = (4,9)	37–79	1H-NMR	Bile (during operation)	6
2018 Wenjie Ma	China	Chinese	17 = (8,11)	42.5±12.3	15 = (8,7)	37.2±8.7	1H-NMR	Bile (post operation)	6
2016 Wenjane Wang	China	Chinese	38 = (14,24)	50.50±18.74	21 = (12,9)	46.30±10.52	RP-HPLC-UV	Bile (during operation)	5
2011 Xia Xu	China	Chinese	15 = (10,5) and 33 = (16,17) ^e^	56.5(29–84) and 57(28–87) ^e^	10 = (7,3)	69.5(53–86)	UPLC-MS	Bile (during operation)	5
2005 Bin Miao	China	Chinese	41 = (16,25)	56±10	19 = (9,10)	53.1±13	RP-HPLC	Bile (during operation)	5
2003 Jinpeng Chen	China	Chinese	100 = (47,53)	50.35	50 = (29,21) *	61.1	HPLC	Bile (during operation)	4
2001 M Fracchia	Italy	English	20 = (10,10)	54(34–77)	17 = (10,7) *	41(21–62)	HPLC	Bile (fasting)	5
1997 Zhiyong Dai	China	Chinese	30	NG	20 *	NG	HPLC	Bile	3
1993 David W. Hay	America	English	29	NG	10 *	NG	HPLC	Bile (during operation)	3
1992 Saixiong Tong	China	Chinese	27	NG	10 *	NG	RP-HPLC	Bile	4
1990 Shaogao Liu	China	Chinese	23	NG	7 *	NG	HPLC	Bile	3
1973 M.M. Fisher ^b^	Canada	English	43 = (20,23)	52(men),50(women)	40 = (20,20) *	47(men),40(women)	GC	Bile (during operation)	4
2022 Zhiyuan Hao ^b^	China	English	19 = (8,11)	63(27–73)	19 = (8,11)	66 (26–67)	LC-MS	Stool (Morning)	9
2015 Nirit Keren	Israel	English	14 = (5,9)	61±9.6	16 = (6,10) *	62.7±11.1	GC-LC	Stool	5
1999 Arnaldo Mamianetti	Argentina	English	10 = (4,6)	54.6(40.69)	10 = (5,5) *	43.7 (35–48)	RP-HPLC	Stool	4

Note:

^a^ Gallstones with cholesterol polyp;

^b^ Gallstones with chronic cholecystitis;

^c^ GC equipped with electron capture detector;

^d^ HPLC and fluorescence detector;

^e^ Sample size of cholecystolithiasis and choledocholithiasis.

NG: Not given.

* Significant different in age and sex or no mention

*Abbreviation*: NOS, Newcastle-Ottawa Scale; HC, Healthy controls; I, Individuals without GSD; P, Patients without GSD and with other diseases; D, Liver donors and hepatic tumor patients without GSD; 1H-NMR, Nuclear magnetic resonance; GC, Gas chromatography; GC-MS, Gas chromatography-mass spectrometry; HPLC, high performance liquid chromatography; HPLC/MS/MS, high performance liquid chromatography coupled with tandem mass spectrometry; HPLC-MS, high performance liquid chromatography-mass spectrometry; LC-MS, Liquid chromatography-mass spectrometry; RP-HPLC, Reversed phase-high performance liquid chromatography; RP-HPLC-UV, Reversed phase-high performance liquid chromatography-ultraviolet detection; UPLC-ESI-MS/MS, Ultra performance liquid chromatography-electrospray ionization-mass spectrometry; UPLC-MS, Ultra performance liquid chromatography-mass spectrometry; UPLC-MS/MS, Ultra performance liquid chromatography-mass spectrometry/mass spectrometry; UPLC-Q-TOF-MS, Ultra high performance liquid chromatography-quadrupole time-of-flight mass spectrometry.

A total of 39 BAs or their ratios in 30 studies were extracted and entered the qualitative review. Thereinto, 17 species of BAs were reported with accurate concentrations over 2 times in 16 studies [19, 29, 35, 37–41, 46, 48–54], which made the meta-analysis feasible. Sixteen studies measured the BAs by using GC [35, 37], high performance liquid chromatography (HPLC) [48, 51, 52, 54], reversed phase-high performance liquid chromatography (RP-HPLC) [49, 50, 53], LC-MS [19], high performance liquid chromatography-mass spectrometry (HPLC-MS) [40], and ultra performance liquid chromatography-mass spectrometry (UPLC-MS) [29, 38, 39, 41, 46], and identified BAs by internal standards.

The concentration of BAs from 16 studies were all reported with absolute quantification method, and the studies with relative quantification were all deleted when performing the meta-analysis. The unit transformation was applied in 6 studies [19, 38, 39, 41, 46, 54]. In 13 studies the concentration data were directly obtained from the article, and in the other 3 studies [19, 29, 39], the data were extracted by software from the box plots. Median with quartiles or interquartile range was transformed to mean and standard deviation in 1 studies [38] and 3 studies [19, 39, 41], respectively, which accounted for 38% of the total synthesized data. Thirty-seven percent of the concentration data presented skewed distribution, which might cause some loss of statistical efficiency in our combined results. The specific characteristics of these BAs are shown in S4 Table.

### Risk of bias

The quality of the 30 studies were assessed by using the NOS. Three studies scored over 7, and 19 studies scored 6 and 5; the remaining 8 studies scored 4 and 3 which were deemed to be low quality. The main factor affecting the quality of studies was the comparability between case and control. The high-quality studies controlled not only the age and sex, but also clinical indicators, such as BMI, aspartate aminotransferase (AST) and alanine aminotransferase (ALT). The NOS scores and details of quality assessment are shown in Table 1 and S5 Table, respectively.

### Qualitative review

**Fig 2:**
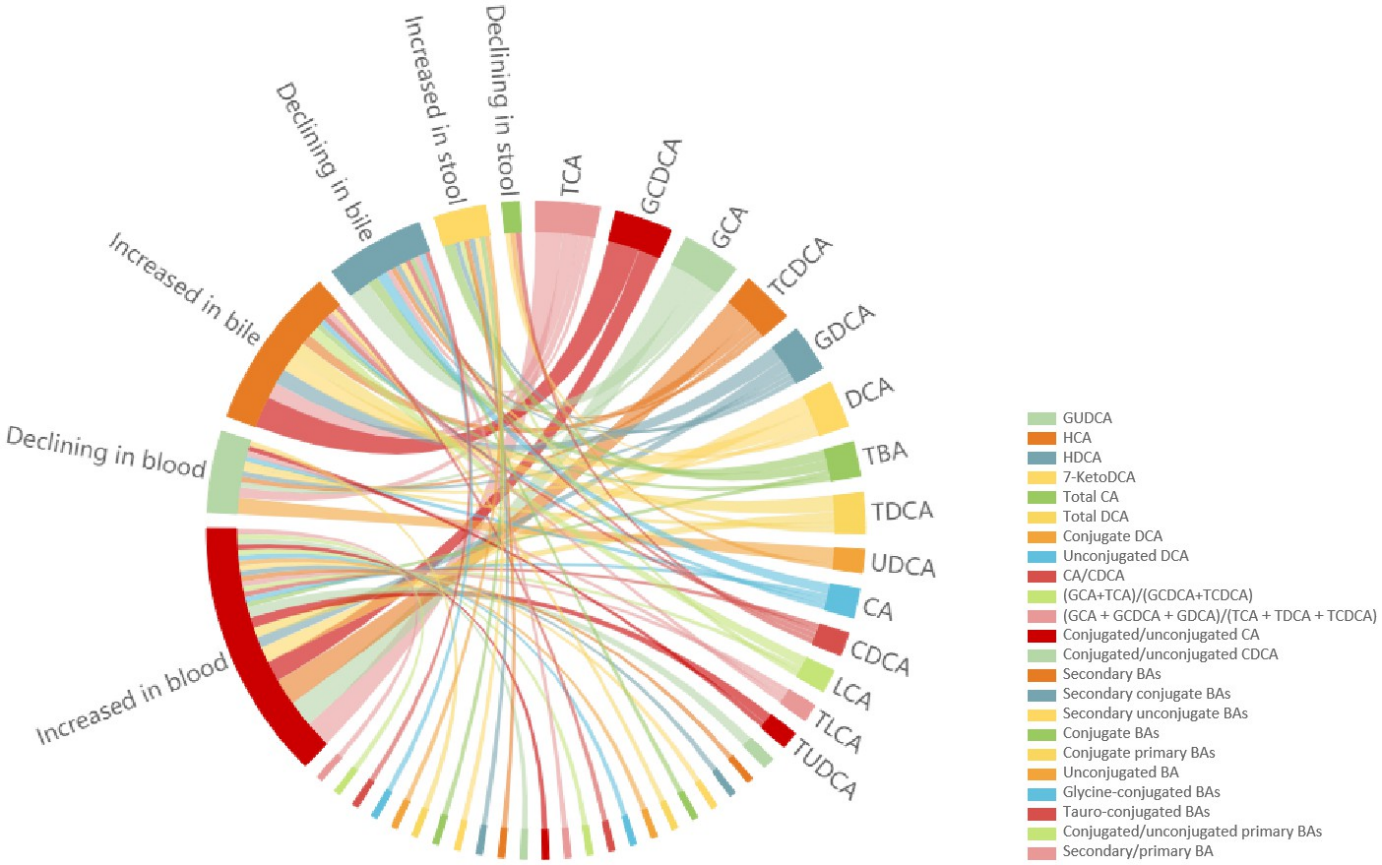
The qualitative review of significant changed bile acids between case group and control group in different biological samples.

Thirty-nine BAs and their ratios were significantly different between case group and control group in diverse biological samples (Fig 2 and S6 Table). Blood samples were the most frequently used form of samples for detection, and the result showed that serum Taurocholic Acid (TCA), Glycochenodeoxycholic acid (GCDCA), Glycocholic acid (GCA), Taurochenodeoxycholic acid (TCDCA), Glycodeoxycholic acid (GDCA) and Deoxycholic acid (DCA) were increased, yet UDCA was decreased in case group. In bile samples, TCA, GCDCA, GDCA, DCA and Taurodeoxycholic acid (TDCA) raised and GCA declined in case group. Fecal samples were seldom used to measure BA profile, the existing studies only indicated that fecal Total BAs (TBA) was elevated in GSD patients. Other BAs and the ratios were rarely reported and their trends were contradictory in different studies. In addition, one study [30] demonstrated the primary BAs and the ratio of glycine and taurine conjugated BAs were significantly different in GSD patients compared to the control subjects, but the trends were not reported, which led to their absence in the Fig 2.

### Meta analysis

**Fig 3:**
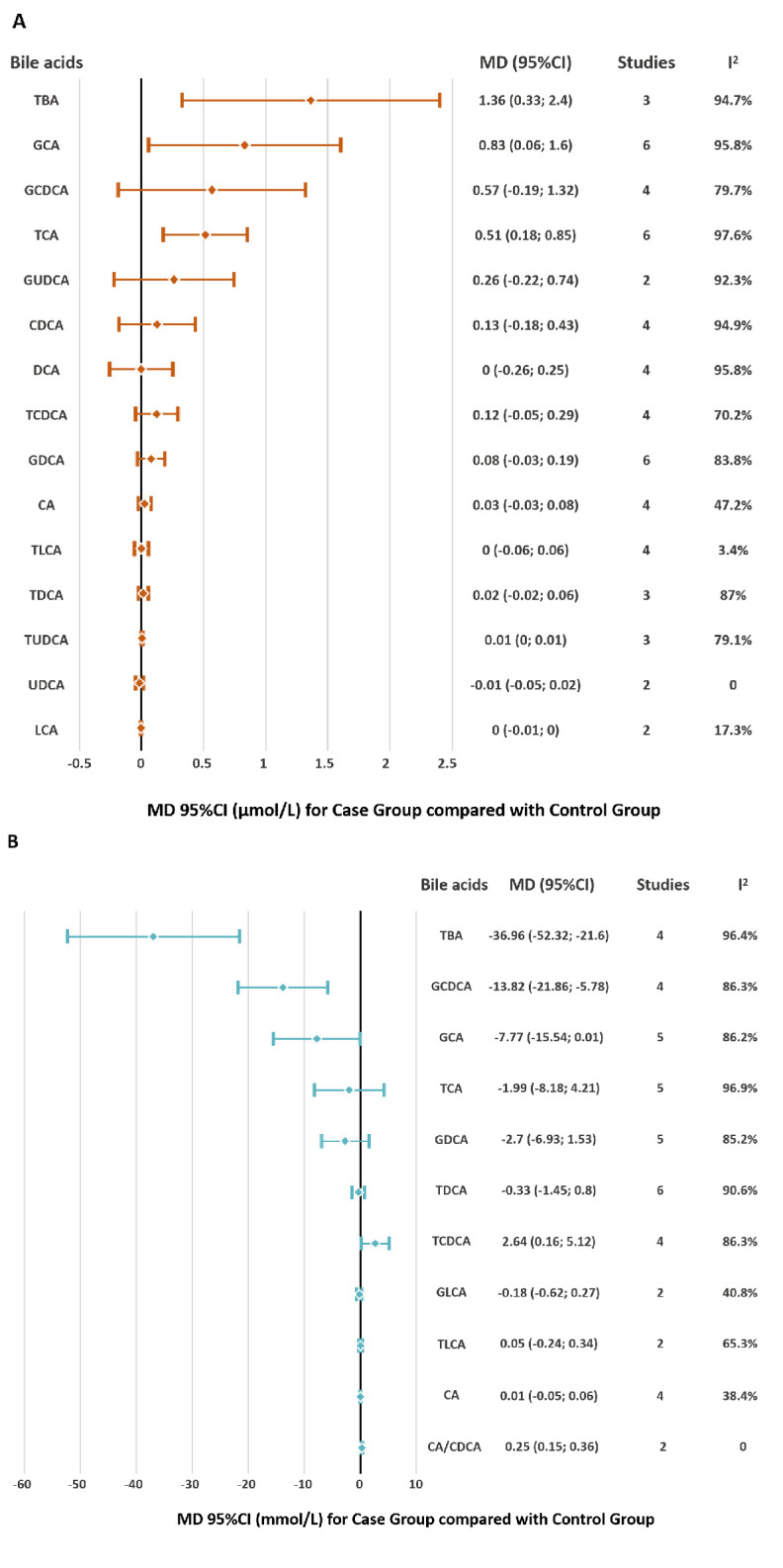
Pooled estimates of bile acids concentration compared between disease and control groups. Overall estimates obtained from forest plots. (A) Serum; (B) Bile.

Meta analysis demonstrated that in GSD patients, serum TBA elevated significantly (WMD = 1.36μmol/L, 95%CI = 0.33; 2.4), yet biliary TBA declined (WMD = -36.96mmol/L, 95%CI = -52.32; -21.6). Both serum GCA (WMD = 0.83μmol/L, 95%CI = 0.06; 1.6) and TCA (WMD = 0.51μmol/L; 95%CI = 0.18; 0.85) increased. Biliary TCDCA (WMD = 2.64mmol/L, 95%CI = 0.16; 5.12) and the ratio of Cholic Acid (CA) and Chenodeoxycholic acid (CDCA) (WMD = 0.25, 95%CI = 0.15; 0.36) increased; biliary GCDCA (WMD = -13.82mmol/L, 95%CI = -21.86; -5.78) fells down. The results of meta-analysis are shown in Fig 3.

Notably, substantial heterogeneity was detected in all comparisons. However, the sensitivity analysis did not show significant difference compared with previous results. Statistical differences were only detected for 3 species of BAs between case and control groups after the high-risk studies have been removed (Table 2). I^2^ of all species of BAs was over 30%, and we regarded that the heterogeneity was mainly derived from 3 studies [50, 52, 54], which applied HPLC to analyzes the sample and were published before 2000. We didn’t assess publication bias due to the insufficient data.

**Table 2:** The statistically significant weighted MD of BAs after sensitivity analysis.

BAs	Sample	Omitting Studies	Studies for Synthesis	WMD	95%-CI	I^2^
GCDCA	Serum	Omitting Xueping Ma-1995	3	0.87	[0.23; 1.52]	86.30%
GCA	Bile	Omitting Shaogao Liu-1990 and Jinpeng Chen-2003	3	-4.66	[-8.68, -0.64]	16%
GDCA	Bile	Omitting Shaogao Liu-1990	4	-4.18	[-8.29; -0.08]	92.90%

Note: MD (μmol/L) for serum, MD (mmol/L) for bile.

*Abbreviation*: GCDCA, Glycochenodeoxycholic Acid; GCA, Glycocholic Acid; GDCA, Glycodeoxycholic Acid.

### Subgroup analysis

**Fig 4:**
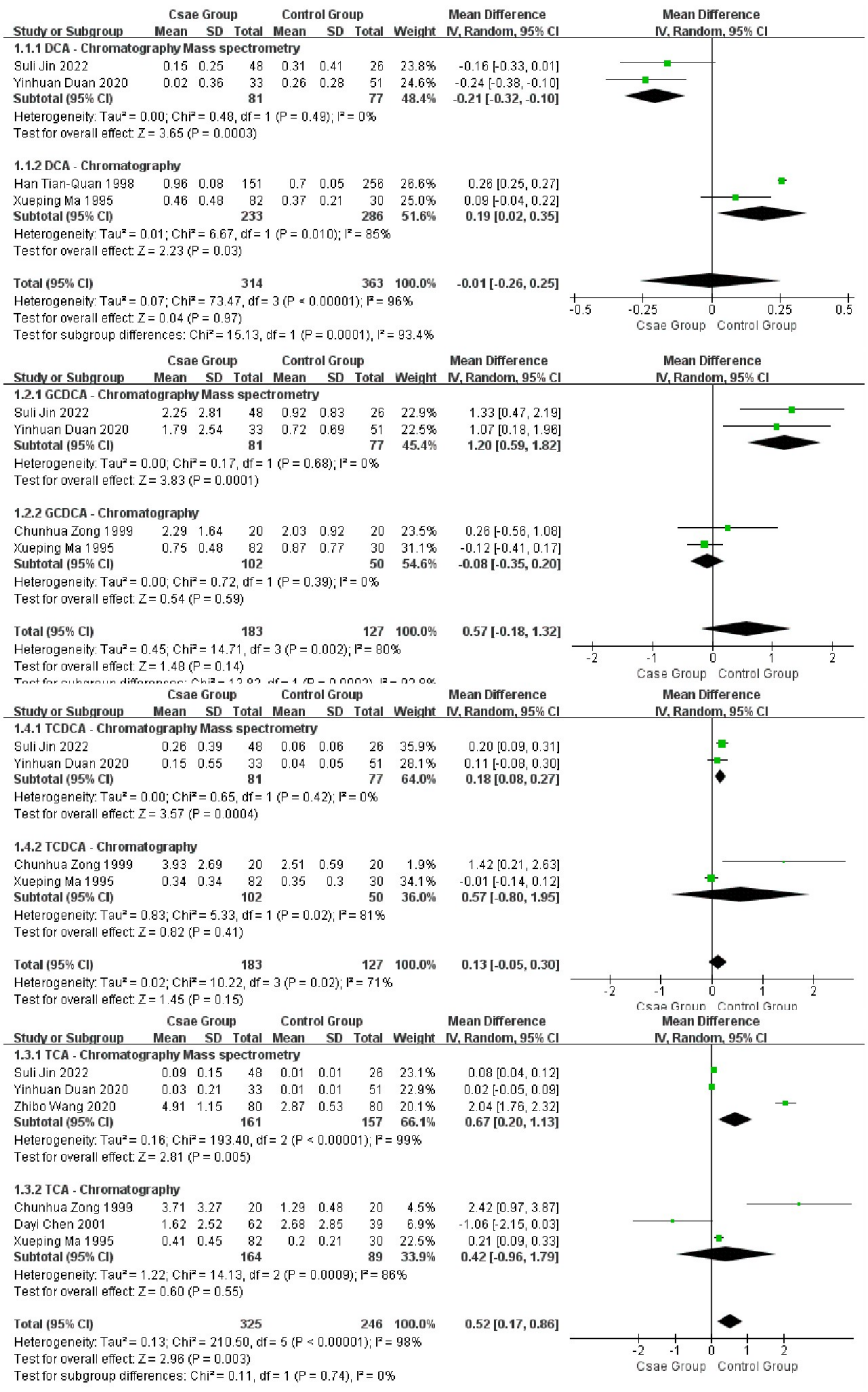
Forest plots of subgroup analysis based on metabolomics technology in serum sample.

Subgroup analysis was feasible for 4 serum BAs in 7 studies [35, 38, 40, 41, 49, 50, 52] based on metabolomics technology. Among the 7 studies, 3 studies [38, 40, 41] applied chromatography mass spectrometry, the other 4 studies [35, 49, 50, 52] used only chromatography method, and all 7 studies identified BAs by internal standards. Meanwhile, the publish years were also polarized, as the former 3 studies were published from the year 2020 to 2022, whereas the latter 4 studies from 1995 to 2001. The subgroup analysis demonstrated completely contradictory results in the changes of DCA level by using the two different technologies. In addition, GCDCA and TCDCA were rising with low heterogeneity yet TCA was growing slightly with high heterogeneity in GSD patients, these distinct differences were only detected by using the LC-MS (Fig 4).

## Discussion

Our study provided a systematic overview on the BA profiles in GSD patients via metabolomics approaches. In GSD patients, biliary TBA decreased yet serum and fecal TBA increased, which serum GCA, TCA and GCDCA elevated significantly in GSD patients. In addition, serum TCDCA, GCDCA, DCA, TDCA, GDCA and UDCA also altered. Considering the serum biomarkers for the early detection has multiple advantages in terms of ease of sampling, applicability, and cost-effectiveness in terms of patient acceptance. These serum BAs can serve as suitable candidates for early warning signs, which is possible to stratify an individual’s risk of developing GSD, thereby identifying high-risk groups that require closer monitoring or early intervention. Subsequently, gallstone formation could be prevented through dietary changes, increased physical activity or the use of pharmacological interventions. Thus, our findings will contribute to the early detection and primary prevention of GSD.

### BA metabolism

BAs are synthesized form cholesterol in hepatocyte by the classical (neutral) synthesis pathway and alternative (acidic) pathways [56]. 7α-hydroxylase (CYP7A1) initiates the classical pathway and is the only rate-limiting enzyme in BA synthesis, which synthesizes two primary BAs, CA and CDCA, then they are amino conjugated with glycine or taurine [57]. Conjugated BAs are secreted into bile and stored in the gallbladder, then participate in the enterohepatic circulation, being the most important process of BA metabolism. In enterohepatic circulation, BAs are reabsorbed in the intestine, and transported back to the liver by passive transport and various BAs transport proteins. Specially, in the distal intestine, conjugated-CA and CDCA are first deconjugated, and then bacterial 7α-dehydroxylase converts CA and CDCA to DCA and Lithocholic acid (LCA) which are called the secondary BAs, respectively. Then, part of DCA and most LCA are excreted into feces and the rest parts are circulated to the liver [58].

### Dysregulated BAs and gallstone formation

The composition of bile contains not only BAs, but also cholesterol, phospholipids, bilirubin, fatty acids, vitamins and minerals [59]. Cholesterol supersaturation in gallbladder bile is the major contributing factor to the formation of gallstone, resulting from abnormal BA synthesis and transport [60, 61], which cholesterol in bile cannot maintain the micelles state and then crystallizes to form cholesterol stones [62]. Previous study has established the correlation between dysregulation of BAs and increased biliary cholesterol secretion [63]. A Swedish study showed that the shortage of BAs was a major cause of supersaturated bile with cholesterol in GSD patients [64]. A clinical study illustrated that female GSD patients showed a significantly smaller BA pool size with an enhanced CA to DCA biotransformation when compared to the healthy female controls [65]. Another study showed that hydrophobic DCA promotes cholesterol crystallization [61],which was consistent with the results of the subgroup analyses in our study. However, the subgroup analysis also showed that the changing trends of DCA were significantly opposite under different techniques of detection, reflecting impressive technological advances. It also suggests that the specificity and sensitivity for detectability of DCA were susceptible to a variety of factors such as the detecting technique, as well as sample handling and preparation.

Our findings showed that biliary TBA decreased yet serum and fecal TBA increased, and conjugated BAs, especially GCA, TCA and GCDCA, elevated in GSD patients. These changes of BAs were mainly caused by abnormal synthesis and transport of BAs. In terms of BA synthesis, CYP7A1 is synchronously inhibited by small heterodimer partner (SHP), a downstream target of FXR in the liver, and fibroblast growth factor 19 (FGF19), which was secreted by FXR in enterocytes [66]. The inactivation of CYP7A1 contributed to gallstone formation by decreasing the production of BAs and increasing cholesterol accumulation [67, 68]. Moreover, pregnane X receptor, and vitamin D receptor were also implicated in GSD by repressing CYP7A1 [69, 70]. Furthermore, BA transports were also impaired, which led to BAs elevation in blood and faeces. A genome-wide association meta-analysis demonstrated that lower apical sodium-dependent BA transporter (ASBT), the BAs transporter which took responsibility for about 95% BAs effectively resorbed at the level of the distal ileum, was accompanied by greater risk of GSD [71]. The abnormally expressed of ASBT was caused by two distinct low frequency missense variants in SLC10A2, which leaded more BAs to be excreted into feces [71]. Another study demonstrated ASBT expression was significantly diminished in the non-overweight GSD carriers compared to non-overweight controls [72]. The impairment of organic solute transporters alpha and beta, the ileal BA transporters, could also lead to low ileal BA reabsorption and an altered BA pool composition, which contributed to the formation of gallstone in non-obese GSD patients [73]. Hepatic sodium/BA cotransporter was the main transporter protein for BAs absorbed in the liver, whose inhibition accelerated the rise of serum BAs [74].

### Intestinal microflora facilitates gallstone formation by effecting homeostasis of BAs

Microflora has an extremely significant effect on the metabolism of BAs, and is also involved in GSD not only by regulating BA receptors, but also by directly converting BAs to hydrophobic derivatives. Increased H_2_S, the metabolic product of *Desulfovibrionales*, induced hepatic FXR and inhibited CYP7A1 expression [75]. It was reported that *Lactobacillus* might relieve cholesterol gallstone through the FXR signaling pathways [76]. L-Theanine, a biologically active ingredient in tea, decreased the activity of bile-salt hydrolase and increased the levels of ileum conjugated BAs by modulating the gut microbiota, resulting in inhibition of intestinal FXR-fibroblast growth factor 15 (FGF15) signaling pathway and upregulation of CYP7A1 [77]. One research discovered fecal transplantation of gut microbiota from GSD patients to gallstone-resistant strain of mice can induce gallstone formation. The transplantation enhanced cecal secondary BAs production and increased BA hydrophobicity, which facilitated intestinal cholesterol absorption [75]. Dysbacteriosis made more hydrophilic primary BAs convert into more hydrophobic and toxic secondary BAs via 7α-dehydroxylase activity, and conjugated BAs to toxic free BAs by bile salt hydrolase activity, while those change can be reversed to protect against gallstone via bacterial hydroxysteroid dehydrogenase activity [61]. Animal studies have confirmed that dietary supplementation with *Lactobacillus* can reduce the relative abundance of *Clostridium*, production of LCA and DCA, and cholesterol consumption [78].

### BAs receptor FXR and gallstone formation

FXR was the primary regulator of BA homeostasis, which negatively regulated the hepatic BA pool by reducing *de novo* synthesis and reabsorption, and enhancing BA export into circulation [61]. As mentioned earlier, FXR promoted stone formation by inhibiting CYP7A1, which subsequently leaded to decrease of BAs production and increase of cholesterol accumulation. CDCA, CA, DCA, and LCA were all FXR agonists that can activate the FXR-CYP7A1 signaling pathway [79], which inhibited cholesterol consumption and causes abnormal cholesterol levels. The FXR agonist obeticholic acid treatment has been found to increase the risk of gallstone formation by triggering relaxation and filling of the gallbladder, increasing cholesterol saturation and BAs hydrophobicity [80]. Knockout of intestinal FXR markedly increased levels of the biliary cholesterol, BAs, and phospholipids in gallbladder bile, which led to reduced Cholesterol saturation index (CSI); Tauroursodeoxycholic acid, the intestinal-specific FXR antagonist, which inhibited the expression of FGF15 and SHP, was also observed to decrease CSI. These results suggested that inhibition of intestinal FXR reduced gallstone formation [81]. However, another study showed that whole-body knockout of FXR sensitized mice to cholesterol gallstone by decreasing the expression of BA export pump (BSEP), reducing the secretion of BAs, and thereby increasing the CSI [82]. As inhibition of intestinal FXR had no effect on the expression of BSEP, liver and intestinal FXR may have different roles in bile secretion and gallstone formation [81]. In addition, FXR is also a critical regulator of normal cholesterol metabolism, which was proven to increase biliary cholesterol elimination in FXR-deficient mice [83].

### Limitations and prospections

It cannot be ignored that the heterogeneity of the meta-analysis was high. The reason is considered that BAs are downstream metabolites, and the stability and reliability of the assay results are related to a variety of factors such as assay technology. Therefore, future validation in larger multicenter samples is needed. In addition, BA profile is associated with gender, age, and BMI. A study based on healthy individuals showed, firstly, that TBA was significantly higher in men than in women; secondly, that TBA was significantly higher in obese women than in lean women; and thirdly, that the difference of BA profiles between male and female subjects decreased at the age of 50–70 years, whereas increased between lean and obese subjects at the same age [84]. However, further analyses and evaluations could not be carried out because the literature included in this paper did not contain data on the effects of repeated testing and diet in the same patients. Prospective cohort studies are needed in future to explore BAs biomarkers with reliability and stability; and more comprehensive multifactorial analyses including gender, age, genetic, metabolic, dietary, and lifestyle factors are also needed to better understand the factors influencing BA profiles and BAs predictive ability.

## Conclusion

Our study provided a comprehensive overview and summary of the BAs profiles in GSD patients by using metabolomics approaches in clinical studies. These findings further corroborated the contribution of dysregulated BAs pathophysiologic to gallstone formation. Our study confirmed that the detection of BAs via metabolomics could provide important prognostic information in GSD patients, which might facilitate the earlier detection and management of GSD and will be beneficial for the development of drugs for GSD treatment.

## Supporting information

S1 Table Search strategy. (PDF)

S2 Table The molar mass of each bile acid for transforming unit. (PDF)

S3 Table Formula 1 for converting median and interquartile range (IQR) into mean and standard deviation (SD). (PDF)

S4 Table The characteristics for each bile acids which performed the meta-analysis. (PDF)

S5 Table The detail of quality assessment and NOS score. (PDF)

S6 Table The frequency of significant changed bile acids between case group and control group in different biological samples. (PDF)

## Abbreviations

95% CI: 95% Confidence interval
ALT: Alanine aminotransferase
ASBT: Apical sodium-dependent bile acid transporter
AST: Aspartate aminotransferase
BAs: Bile acids
BMI: Body mass index
BSEP: Bile acid export pump
CA: Cholic acid
CDCA: Chenodeoxycholic acid
CSI: Cholesterol saturation index
CYP7A1: 7α-Hydroxylase
DCA: Deoxycholic acid
FGF15: Fibroblast growth factor 15
FGF19: Fibroblast growth factor 19
FXR: Farnesoid X receptor
GC: Gas chromatography
GCA: Glycocholic acid
GCDCA: Glycochenodeoxycholic acid
GC-MS: Gas chromatography-mass spectrometry
GDCA: Glycodeoxycholic acid
GSD: Gallstone disease
HPLC: High performance liquid chromatography
HPLC-MS: High performance liquid chromatography-mass spectrometry
LCA: Lithocholic acid
LC-MS: Liquid chromatography-mass spectrometry
NMR: Nuclear magnetic resonance
RP-HPLC: Reversed phase-high performance liquid chromatography
SHP: Small heterodimer partner
TBA: Total bile acid
TCA: Taurocholic acid
TCDCA: Taurochenodeoxycholic acid
TDCA: Taurodeoxycholic acid
TG: Triglyceride
UDCA: Ursodeoxycholic acid
UPLC-MS: Ultra performance liquid chromatography-mass spectrometry
WMDs: weighted mean differences
