# Supplementary material for: Predictive value of bile acids as metabolite biomarkers for gallstone disease: A systematic review and meta-analysis
Source: PLoS One. 2024 Jul 25;19(7):e0305170. doi: 10.1371/journal.pone.0305170 (PMC11271903; doi:10.1371/journal.pone.0305170)
Supplement: S1 Table — (PDF) [file pone.0305170.s001.pdf]

S1 Table Search strategy

PubMed	#1	((("Gallstones"[Mesh]) OR ((((((((((((((Gallstone[Title/Abstract]) OR (Gall Stones[Title/Abstract])) OR (Gall Stones[Title/Abstract])) OR (Biliary Calculi[Title/Abstract])) OR (Calculi, Biliary[Title/Abstract])) OR (Gall Stone[Title/Abstract])) OR (Common Bile Duct Calculi[Title/Abstract])) OR (Biliary Calculi, Common Bile Duct[Title/Abstract])) OR (Gallstones, Common Bile Duct[Title/Abstract])) OR (Common Bile Duct Gall Stone[Title/Abstract])) OR (Common Bile Duct Gallstones[Title/Abstract])) OR (Gall Stones, Common Bile Duct[Title/Abstract])) OR (Common Bile Duct Gallstone[Title/Abstract])) OR (Common Bile Duct Gall Stones[Title/Abstract]))) OR (((((("Cholelithiasis"[Mesh]) OR (((Cholelithiases[Title/Abstract]) OR (Gallstone Disease[Title/Abstract])) OR (Gallstone Diseases[Title/Abstract]))) OR (("Cholelithiasis"[Mesh]) OR (((Cholelithiases[Title/Abstract]) OR (Gallstone Disease[Title/Abstract])) OR (Gallstone Diseases[Title/Abstract]))))) OR ("cholecystolithiasis"[MeSH])) OR ("choledocholithiasis"[MeSH])) OR (Common Bile Duct[Title/Abstract]))) OR (((((((((((((((((((cholesterol stone[Title/Abstract]) OR (pigment stone[Title/Abstract])) OR (bile cholelith[Title/Abstract])) OR (bile lithiasis[Title/Abstract])) OR (bile stone*[Title/Abstract])) OR (bilestone*[Title/Abstract])) OR (biliary calculus[Title/Abstract])) OR (biliary cholelith[Title/Abstract])) OR (biliary lithiasis[Title/Abstract])) OR (biliary stone*[Title/Abstract])) OR (black pigment stone*[Title/Abstract])) OR (cholecystectomy[Title/Abstract])) OR (gall cholelith[Title/Abstract])) OR (gall lithiasis[Title/Abstract])) OR (gall* stone*[Title/Abstract])) OR (gallbladder cholelith[Title/Abstract])) OR (gallbladder lithiasis[Title/Abstract])) OR (gallbladder stone*[Title/Abstract])) OR (gallstone*[Title/Abstract]))
	#2	(((("Metabolomics"[Mesh]) OR ((((Metabolomic[Title/Abstract]) OR (Metabonomics[Title/Abstract])) OR (Metabonomic[Title/Abstract]))) OR (((((((((((((((((((((((((((((Metabolomic*[Title/Abstract]) OR (Metabonomic*[Title/Abstract])) OR (Metabolit*[Title/Abstract])) OR (H NMR[Title/Abstract])) OR (nuclear magnetic resonance spectroscopy[Title/Abstract])) OR (proton NMR[Title/Abstract])) OR (proton nuclear magnetic resonance[Title/Abstract])) OR (gas chromatogra*[Title/Abstract])) OR (GC-MS[Title/Abstract])) OR (gas chromatograph-mass spectrometry[Title/Abstract])) OR (GC-TOF-MS[Title/Abstract])) OR (gas chromatography/time-of-flight mass spectrometry[Title/Abstract])) OR (liquid chromatogra*[Title/Abstract])) OR (LC-MS[Title/Abstract])) OR (liquid-chromatography mass spectrometry[Title/Abstract])) OR (TQ MS[Title/Abstract])) OR (triple quadrupole mass spectrometry[Title/Abstract])) OR (UPLC[Title/Abstract])) OR (ultra performance liquid chromatograph*[Title/Abstract])) OR (ultra-performance liquid chromatograph*[Title/Abstract])) OR (HPLC[Title/Abstract])) OR

		(high performance liquid chrormatograph*[Title/Abstract])) OR (high-performance liquid chrormatograph*[Title/Abstract])) OR (UHPLC-MS/MS[Title/Abstract])) OR (ultra-high performance liquid chromatography/tandem mass spectrometry[Title/Abstract])) OR (UPLC-QTOF-MS[Title/Abstract])) OR (ultraperformance liquid chromatography quadruple time-of-flight mass spectrometer[Title/Abstract])) OR (UHPLC-TQ-MS[Title/Abstract])) OR (ultra-high performance liquid chromatography triple quadrupole mass spectrometry[Title/Abstract]))) OR (("Metabolomics"[Mesh]) OR ((((Metabolomic[Title/Abstract]) OR (Metabonomics[Title/Abstract])) OR (Metabonomic[Title/Abstract]))) OR (((((((((((((((((((((((((((Metabolomic*[Title/Abstract]) OR (Metabonomic*[Title/Abstract])) OR (Metabolit*[Title/Abstract])) OR (H NMR[Title/Abstract])) OR (nuclear magnetic resonance spectroscopy[Title/Abstract])) OR (proton NMR[Title/Abstract])) OR (proton nuclear magnetic resonance[Title/Abstract])) OR (gas chromatogra*[Title/Abstract])) OR (GC-MS[Title/Abstract])) OR (gas chromatograph-mass spectrometry[Title/Abstract])) OR (GC-TOF-MS[Title/Abstract])) OR (gas chromatography/time-of-flight mass spectrometry[Title/Abstract])) OR (liquid chromatogra*[Title/Abstract])) OR (LC-MS[Title/Abstract])) OR (liquid-chromatography mass spectrometry[Title/Abstract])) OR (TQ MS[Title/Abstract])) OR (triple quadrupole mass spectrometry[Title/Abstract])) OR (UPLC[Title/Abstract])) OR (ultra performance liquid chromatograph*[Title/Abstract])) OR (ultra-performance liquid chromatograph*[Title/Abstract])) OR (HPLC[Title/Abstract])) OR (high performance liquid chrormatograph*[Title/Abstract])) OR (high-performance liquid chrormatograph*[Title/Abstract])) OR (UHPLC-MS/MS[Title/Abstract])) OR (ultra-high performance liquid chromatography/tandem mass spectrometry[Title/Abstract])) OR (UPLC-QTOF-MS[Title/Abstract])) OR (ultraperformance liquid chromatography quadruple time-of-flight mass spectrometer[Title/Abstract])) OR (UHPLC-TQ-MS[Title/Abstract])) OR (ultra-high performance liquid chromatography triple quadrupole mass spectrometry[Title/Abstract])))) OR ("Metabolome"[Mesh])) OR (((((Metabolomes[Title/Abstract]) OR (Metabolic Profile[Title/Abstract])) OR (Metabolic Profiles[Title/Abstract])) OR (Profile, Metabolic[Title/Abstract])) OR (Profiles, Metabolic[Title/Abstract]))
	#3	#1 AND #2
	#4	"Animals"[Mesh]
	#5	"Humans"[Mesh]
	#6	#4 NOT #5
	#7	#3 NOT #6
Web of	#1	TS=(cholelithiasis OR Cholelithiases OR Gallstone Disease OR Gallstone Diseases OR cholecystolithiasis OR choledocholithiasis OR

Science		Common Bile Duct OR Gallstones OR Gallstone OR Gall Stones OR Biliary Calculi OR Calculi, Biliary OR Gall Stone OR Common Bile Duct Calculi OR Biliary Calculi, Common Bile Duct OR Gallstones, Common Bile Duct OR Common Bile Duct Gall Stone OR Common Bile Duct Gallstones OR Gall Stones, Common Bile Duct OR Common Bile Duct Gallstone OR Common Bile Duct Gall Stones OR cholesterol stone OR pigment stone OR bile cholelith OR bile lithiasis OR bile stone* OR bilestone* OR biliary calculus OR biliary cholelith OR biliary lithiasis OR biliary stone* OR black pigment stone* OR calculi OR cholecystectomy OR gall cholelith OR gall lithiasis OR gall* stone* OR gallbladder cholelith OR gallbladder lithiasis OR gallbladder stone* OR gallstone*)
	#2	TS=(Metabolomic* OR Metabonomic* OR Metabolit* OR Metabolome OR Metabolomes OR Metabolic Profile OR Metabolic Profiles OR Profile, Metabolic OR Profiles, Metabolic OR H NMR OR nuclear magnetic resonance spectroscopy OR proton NMR OR proton nuclear magnetic resonance OR gas chromatogra* OR GC-MS OR gas chromatograph-mass spectrometry OR GC-TOF-MS OR gas chromatography/time-of-flight mass spectrometry OR liquid chromatogra* OR LC-MS OR liquid-chromatography mass spectrometry OR TQ MS OR triple quadrupole mass spectrometry OR UPLC OR ultra performance liquid chromatograph* OR ultra-performance liquid chromatograph* OR HPLC OR high performance liquid chrormatograph* OR high-performance liquid chrormatograph* OR UHPLC-MS/MS OR ultra-high performance liquid chromatography/tandem mass spectrometry OR UPLC-QTOF-MS OR ultraperformance liquid chromatography quadruple time-of-flight mass spectrometer OR UHPLC-TQ-MS OR ultra-high performance liquid chromatography triple quadrupole mass spectrometry)
	#3	#1 AND #2
	#4	TS=Animal
	#5	TS=Humans
	#6	(#4) NOT #5
	#7	(#3) NOT #6
Embase	#1	'cholelithiasis'/exp
	#2	'bile calculosis':ti,ab,kw OR 'bile duct microlithasis':ti,ab,kw OR 'bile lithiasis':ti,ab,kw OR 'bile lithogenesis':ti,ab,kw OR 'bile lithogenicity':ti,ab,kw OR 'bile microlithiasis':ti,ab,kw OR 'bile stone':ti,ab,kw OR 'bile stone formation':ti,ab,kw OR 'biliary calculi':ti,ab,kw OR 'biliary calculosis':ti,ab,kw OR 'biliary calculous disease':ti,ab,kw OR 'biliary calculus':ti,ab,kw OR 'biliary duct calculus':ti,ab,kw OR 'biliary duct microlithiasis':ti,ab,kw OR 'biliary lithiasis':ti,ab,kw OR 'biliary lithogenesis':ti,ab,kw OR 'biliary lithogenicity':ti,ab,kw OR 'biliary microliths':ti,ab,kw OR 'biliary stone':ti,ab,kw OR 'biliary tract calculi':ti,ab,kw OR 'biliary tract

		calculosis':ti,ab,kw OR 'biliary tract calculous disease':ti,ab,kw OR 'biliary tract calculus':ti,ab,kw OR 'biliary tract lithiasis':ti,ab,kw OR 'biliary tract stone':ti,ab,kw OR 'calcic biliary lithiasis':ti,ab,kw OR 'calcified gallbladder concrements':ti,ab,kw OR 'calcified gallbladder stone':ti,ab,kw OR 'calcified gallbladder stones':ti,ab,kw OR 'calcified gallstones':ti,ab,kw OR 'calcium carbonate gallstone':ti,ab,kw OR 'calcium carbonate stone (cholelithiasis)':ti,ab,kw OR 'calcium cholelithiasis':ti,ab,kw OR 'calcium containing gallbladder calculi':ti,ab,kw OR 'calcium gall stone':ti,ab,kw OR 'calcium gallstone':ti,ab,kw OR 'calcium-containing gall stones':ti,ab,kw OR 'calcium-containing gallstones':ti,ab,kw OR 'cholecystic lithiasis':ti,ab,kw OR 'cholecystolithiasis':ti,ab,kw OR 'choledochal calculi':ti,ab,kw OR 'choledochal calculus':ti,ab,kw OR 'choledochal stone':ti,ab,kw OR 'choledocholithiasis':ti,ab,kw OR 'choledochus calculi':ti,ab,kw OR 'choledochus calculus':ti,ab,kw OR 'choledochus stone':ti,ab,kw OR 'cholelith':ti,ab,kw OR 'choleliths':ti,ab,kw OR 'cholesterol bile calculosis':ti,ab,kw OR 'cholesterol cholelithiasis':ti,ab,kw OR 'cholesterol cholelithogenesis':ti,ab,kw OR 'cholesterol choleliths':ti,ab,kw OR 'cholesterol gallstone':ti,ab,kw OR 'cholethiasis':ti,ab,kw OR 'common bile duct calculi':ti,ab,kw OR 'common bile duct calculus':ti,ab,kw OR 'common biliary duct stone':ti,ab,kw OR 'common duct gallstone':ti,ab,kw OR 'ductus choledochus stone':ti,ab,kw OR 'gall bladder calculi':ti,ab,kw OR 'gall bladder calculus':ti,ab,kw OR 'gall bladder concretions':ti,ab,kw OR 'gall bladder lithiasis':ti,ab,kw OR 'gall bladder microlithiasis':ti,ab,kw OR 'gall bladder stone':ti,ab,kw OR 'gall stone':ti,ab,kw OR 'gall stone formation':ti,ab,kw OR 'gallbladder calculi':ti,ab,kw OR 'gallbladder calculus':ti,ab,kw OR 'gallbladder concrements':ti,ab,kw OR 'gallbladder concretion':ti,ab,kw OR 'gallbladder lithiasis':ti,ab,kw OR 'gallbladder microlithiasis':ti,ab,kw OR 'gallbladder microliths':ti,ab,kw OR 'gallbladder stone':ti,ab,kw OR 'gallstone disease':ti,ab,kw OR 'gallstones':ti,ab,kw OR 'hepatolithiasis':ti,ab,kw OR 'intrahepatic gallstone':ti,ab,kw OR 'liver stone':ti,ab,kw OR 'residual choledochal stone':ti,ab,kw
	#3	#1 OR #2
	#4	'metabolomics'/exp
	#5	'metabolome'/exp
	#6	'metabolic fingerprinting'/exp
	#7	'metabolic fingerprint':ti,ab,kw OR 'metabolic profiling':ti,ab,kw OR 'metabolism fingerprint':ti,ab,kw OR 'metabolism profiling':ti,ab,kw OR 'metabolite fingerprinting':ti,ab,kw OR 'metabolite profiling':ti,ab,kw OR 'metabolites fingerprinting':ti,ab,kw OR 'metabolites profiling':ti,ab,kw OR 'metabolome profiling':ti,ab,kw OR 'metabolomic profiling':ti,ab,kw
	#8	'metabolomic*':ti,ab,kw OR metabonomic*:ti,ab,kw OR metabolit*:ti,ab,kw OR 'h nmr':ti,ab,kw OR 'nuclear magnetic resonance spectroscopy':ti,ab,kw OR 'proton nmr':ti,ab,kw OR 'proton nuclear magnetic resonance':ti,ab,kw OR 'gas chromatogra*':ti,ab,kw OR 'gc

		ms':ti,ab,kw OR 'gas chromatograph-mass spectrometry':ti,ab,kw OR 'gc tof ms':ti,ab,kw OR 'gas chromatography/time-of-flight mass spectrometry':ti,ab,kw OR 'liquid chromatogra*':ti,ab,kw OR 'lc ms':ti,ab,kw OR 'liquid-chromatography mass spectrometry':ti,ab,kw OR 'tq ms':ti,ab,kw OR 'triple quadrupole mass spectrometry':ti,ab,kw OR uplc:ti,ab,kw OR 'ultra performance liquid chromatograph*':ti,ab,kw OR 'ultra-performance liquid chromatograph*':ti,ab,kw OR hplc:ti,ab,kw OR 'high performance liquid chrormatograph*':ti,ab,kw OR 'high-performance liquid chrormatograph*':ti,ab,kw OR 'uhplc-ms/ms':ti,ab,kw OR 'ultra-high performance liquid chromatography/tandem mass spectrometry':ti,ab,kw OR 'uplc qtof ms':ti,ab,kw OR 'ultraperformance liquid chromatography quadruple time-of-flight mass spectrometer':ti,ab,kw OR 'uhplc tq ms':ti,ab,kw OR 'ultra-high performance liquid chromatography triple quadrupole mass spectrometry':ti,ab,kw
	#9	#4 OR #5 OR #6 OR #7 OR #8
	#10	#3 AND #9
	#11	#3 AND #9 AND [embase]/lim AND [humans]/lim AND [english]/lim
Cochrane Library	#1	MeSH descriptor: [Cholelithiasis] explode all trees
	#2	MeSH descriptor: [Cholecystolithiasis] explode all trees
	#3	MeSH descriptor: [Choledocholithiasis] explode all trees
	#4	MeSH descriptor: [Gallstones] explode all trees
	#5	('Cholelithiases'):ti,ab,kw OR ('Gallstone Disease'):ti,ab,kw OR ('Gallstone Diseases'):ti,ab,kw OR ('Cholelithiasis'):ti,ab,kw OR ('Common Bile Duct'):ti,ab,kw OR ('Biliary Calculi’):ti,ab,kw OR (‘Common Bile Duct'):ti,ab,kw OR ('Gall Stones'):ti,ab,kw OR ('Common Bile Duct’):ti,ab,kw OR (‘Common Bile Duct Gallstones’):ti,ab,kw OR (‘Gallstones'):ti,ab,kw OR ('Common Bile Duct’):ti,ab,kw OR (‘Common Bile Duct Calculi’):ti,ab,kw OR (‘Common Bile Duct Gall Stones’):ti,ab,kw OR (‘Common Bile Duct Gall Stone’):ti,ab,kw OR (‘Common Bile Duct Gallstone’):ti,ab,kw OR (‘Gall Stones’):ti,ab,kw OR (‘Gall Stone’):ti,ab,kw OR (‘Gallstone’):ti,ab,kw OR (‘Calculi, Biliary’):ti,ab,kw OR (‘Biliary Calculi'):ti,ab,kw OR ('cholesterol stone'):ti,ab,kw OR ('pigment stone'):ti,ab,kw OR (‘bile cholelith'):ti,ab,kw OR ('bile lithiasis'):ti,ab,kw OR ('bile stone*'):ti,ab,kw OR ('bilestone*'):ti,ab,kw OR ('biliary calculus'):ti,ab,kw OR ('biliary cholelith'):ti,ab,kw OR ('biliary lithiasis'):ti,ab,kw OR ('biliary stone*'):ti,ab,kw OR ('black pigment stone*'):ti,ab,kw OR ('calculi'):ti,ab,kw OR ('cholecystectomy'):ti,ab,kw OR ('gall cholelith'):ti,ab,kw OR ('gall lithiasis'):ti,ab,kw OR ('gall* stone*'):ti,ab,kw OR ('gallbladder cholelith'):ti,ab,kw OR ('gallbladder lithiasis'):ti,ab,kw OR ('gallbladder stone*'):ti,ab,kw OR ('gallstone*'):ti,ab,kw

	#6	#1 OR #2 OR #3 OR #4 OR #5
	#7	MeSH descriptor: [Metabolomics] in all MeSH products
	#8	MeSH descriptor: [Metabolome] explode all trees
	#9	('Metabonomic'):ti,ab,kw OR ('Metabonomics; Metabolomic'):ti,ab,kw OR ('Profiles, Metabolic'):ti,ab,kw OR ('Profile, Metabolic'):ti,ab,kw OR ('Metabolic Profiles'):ti,ab,kw OR ('Metabolic Profile'):ti,ab,kw OR ('Metabolomes'):ti,ab,kww OR ('Metabolomic*'):ti,ab,kw OR ('Metabonomic*'):ti,ab,kw OR ('Metabolit*'):ti,ab,kw OR ('H NMR'):ti,ab,kw OR ('nuclear magnetic resonance spectroscopy'):ti,ab,kw OR ('proton NMR'):ti,ab,kw OR ('proton nuclear magnetic resonance'):ti,ab,kw OR ('gas chromatogra*'):ti,ab,kw OR ('GC-MS'):ti,ab,kw OR ('gas chromatograph-mass spectrometry'):ti,ab,kw OR ('GC-TOF-MS'):ti,ab,kw OR ('gas chromatography/time-of-flight mass spectrometry'):ti,ab,kw OR ('liquid chromatogra*'):ti,ab,kw OR ('LC-MS'):ti,ab,kw OR ('liquid-chromatography mass spectrometry'):ti,ab,kw OR ('TQ MS'):ti,ab,kw OR ('triple quadrupole mass spectrometry'):ti,ab,kw OR ('UPLC'):ti,ab,kw OR ('ultra performance liquid chromatograph*'):ti,ab,kw OR ('ultra-performance liquid chromatograph*'):ti,ab,kw OR ('HPLC'):ti,ab,kw OR ('high performance liquid chrormatograph*'):ti,ab,kw OR ('high-performance liquid chrormatograph*'):ti,ab,kw OR ('UHPLC-MS/MS'):ti,ab,kw OR ('ultra-high performance liquid chromatography/tandem mass spectrometry'):ti,ab,kw OR ('UPLC-QTOF-MS'):ti,ab,kw OR ('ultraperformance liquid chromatography quadruple time-of-flight mass spectrometer'):ti,ab,kw OR ('UHPLC-TQ-MS'):ti,ab,kw OR ('ultra-high performance liquid chromatography triple quadrupole mass spectrometry'):ti,ab,kw OR ('Metabolic Profile'):ti,ab,kw OR ('Metabolic Profiles'):ti,ab,kw OR ('Profile, Metabolic'):ti,ab,kw OR ('Profiles, Metabolic'):ti,ab,kw
	#10	#7 OR #8 OR #9
	#11	#6 AND #10
Wanfang databases	（主题:(胆结石) or 题名或关键词:(胆囊结石) or 题名或关键词:(胆石症) or 题名或关键词:(胆石病) or 题名或关键词:(胆管结石) ）and （主题:(代谢组学) or 题名或关键词:(代谢组) or 题名或关键词:(代谢谱) or 题名或关键词:(代谢轮廓) or 题名或关键词:(H NMR) or 题名或关键词:(核磁共振) or 题名或关键词:(proton NMR) or 题名或关键词:(质子 NMR) or 题名或关键词:(GC-MS) or 题名或关键词:(气相色谱-质谱联用) or 题名或关键词:(GC-TOF-MS)or 题名或关键词:(气相色谱-飞行时间质谱) or 题名或关键词:(LC-MS) or 题名或关键词:(液相色谱-质谱联用) or 题名或关键词:(TQ MS) or 题名或关键词:(三重四极杆液质联用) or 题名或关键词:(UPLC) or 题名或关键词:(超高效液相色谱) or 题名或关键词:(HPLC) or 题名或关键词:(高效液相色谱)or 题名或关键词:(UHPLC-MS/MS) or 题名或关键词:(超高效液相色谱-串联质谱) or 题名或关键词:(UPLC-QTOF-MS) or 题名或关键词:(超高效液相色谱-四极杆-飞行时间串联质谱) or 题名或关键词:(UHPLC-TQ-MS) or 题名或关键词:(超高效液相色谱三重四极杆质谱)）	

<table>
<tr><td>China National Knowledge Infrastructure (CNKI)</td><td colspan="2">(SU %= '胆结石' OR TKA = '胆囊结石' OR TKA = '胆石症' OR TKA = '胆石病' OR TKA = '胆管结石')AND (SU %= '代谢组学' OR TKA = '代谢谱' OR TKA = '代谢轮廓' OR TKA = '代谢组' OR TKA = 'NMR' OR TKA = '核磁共振' OR TKA = 'proton NMR' OR TKA = '质子 NMR' OR TKA = 'GC-MS' OR TKA = '气相色谱-质谱联用' OR TKA = 'GC-TOF-MS' OR TKA = '气相色谱-飞行时间质谱' OR TKA = 'LC-MS' OR TKA = '液相色谱-质谱联用' OR TKA = 'TQ MS' OR TKA = '三重四极杆液质联用' OR TKA = 'UPLC' OR TKA = '超高效液相色谱' OR TKA = 'HPLC' OR TKA = '高效液相色谱' OR TKA = 'UHPLC-MS/MS' OR TKA = '超高效液相色谱-串联质谱' OR TKA = 'UPLC-QTOF-MS' OR TKA = '超高效液相色谱-四极杆-飞行时间串联质谱' OR TKA = 'UHPLC-TQ-MS' OR TKA = '超高效液相色谱三重四极杆质谱')</td></tr>
<tr><td>VIP Information Resource Integration Service Platform (cqVIP)</td><td colspan="2">K=(胆结石 OR 胆囊结石 OR 胆石症 OR 胆石病 OR 胆管结石) AND K=(代谢组学 OR 代谢组 OR 代谢谱 OR 代谢轮廓 OR H NMR OR 核磁共振 OR proton NMR OR 质子 NMR OR GC-MS OR 气相色谱-质谱联用 OR GC-TOF-MS OR 气相色谱-飞行时间质谱 OR LC-MS OR 液相色谱-质谱联用 OR TQ MS OR 三重四极杆液质联用 OR UPLC OR 超高效液相色谱 OR HPLC OR 高效液相色谱 OR UHPLC-MS/MS OR 超高效液相色谱-串联质谱 OR UPLC-QTOF-MS OR 超高效液相色谱-四极杆-飞行时间串联质谱 OR UHPLC-TQ-MS OR 超高效液相色谱三重四极杆质谱)</td></tr>
<tr><td rowspan="6">China Biology Medicine Disc (SinoMed)</td><td>#1</td><td>"胆结石"[不加权:扩展]</td></tr>
<tr><td>#2</td><td>"胆结石"[常用字段:智能] OR "胆囊结石"[常用字段:智能] OR "胆石病"[常用字段:智能] OR "胆石症"[常用字段:智能] OR "胆管结石"[常用字段:智能]</td></tr>
<tr><td>#3</td><td>#1 OR #2</td></tr>
<tr><td>#4</td><td>"代谢组学"[不加权:扩展]</td></tr>
<tr><td>#5</td><td>"代谢组学"[常用字段:智能] OR "代谢组"[常用字段:智能] OR "代谢谱"[常用字段:智能] OR "代谢轮廓"[常用字段:智能]</td></tr>
<tr><td>#6</td><td>"H NMR"[常用字段:智能] OR "核磁共振"[常用字段:智能] OR "proton NMR"[常用字段:智能] OR "质子 NMR"[常用字段:智能] OR "GC-MS"[常用字段:智能] OR "气相色谱-质谱联用 "[常用字段:智能] OR "GC-TOF-MS"[常用字段:智能] OR "气相色谱-飞行时间质谱"[常用字段:智能] OR "LC-MS OR 液相色谱-质谱联用"[常用字段:智能] OR "TQ MS"[常用字段:智能] OR "三重四极杆液质联用"[常用字段:智能] OR "UPLC"[常用字段:智能] OR "超高效液相色谱"[常用字段:智能] OR "HPLC"[常用字段:智能] OR "高效液相色谱"[常用字段:智能] OR "UHPLC-MS/MS"[常用字段:智能] OR "超高效液相色谱-串联质谱"[常用字段:智能] OR "UPLC-QTOF-MS"[常用字段:智能] OR "超高效液相色谱-四极杆-飞行时间串联质谱"[常用字段:智能] OR "UHPLC-TQ-MS"[常用字段:智能] OR "超高效液相色谱三重四极杆质谱"[常用字段:智能]</td></tr>
</table>

	#7	#4 OR #5 OR #6
	#8	#3 AND #7
	#9	#8 AND ("临床试验"[文献类型])
