# Supplementary material for: Predictive value of bile acids as metabolite biomarkers for gallstone disease: A systematic review and meta-analysis
Source: PLoS One. 2024 Jul 25;19(7):e0305170. doi: 10.1371/journal.pone.0305170 (PMC11271903; doi:10.1371/journal.pone.0305170)
Supplement: S2 Table — (PDF) [file pone.0305170.s002.pdf]

S2 Table The molar mass of each bile acid for transforming unit.

BAs	PubChem CID	molecular formula	Molecular Weight (g/mol)
CA	221493	C24H40O5	408.6
CDCA	10133	C24H40O4	392.6
DCA	222528	C24H40O4	392.6
GCA	10140	C26H43NO6	465.6
GCDCA	12544	C26H43NO5	449.6
GDCA	3035026	C26H43NO5	449.6
GLCA	115245	C26H43NO4	433.6
GUDCA	12310288	C26H43NO5	449.6
LCA	9903	C24H40O3	376.6
TCA	6675	C26H45NO7S	515.7
TCDCA	387316	C26H45NO6S	499.7
TDCA	2733768	C26H45NO6S	499.7
TLCA	439763	C26H45NO5S	483.7
TUDCA	9848818	C26H45NO6S	499.7
UDCA	31401	C24H40O4	392.6

Abbreviations: CA, Cholic Acid; CDCA, Chenodeoxycholic Acid; DCA, Deoxycholic Acid; GCA, Glycocholic Acid; GCDCA, Glycochenodeoxycholic Acid; GDCA, Glycodeoxycholic Acid; GLCA, Glycolithocholic Acid; GUDCA, Glycoursodeoxycholic Acid; LCA, Lithocholic Acid; TCA, Taurocholic Acid; TCDCA, Taurochenodeoxycholic Acid; TDCA, Taurodeoxycholic Acid; TLCA, Taurolithocholic Acid; TUDCA, Tauroursodeoxycholic Acid; UDCA, Ursodeoxycholic.
