# Supplementary material for: Predictive value of bile acids as metabolite biomarkers for gallstone disease: A systematic review and meta-analysis
Source: PLoS One. 2024 Jul 25;19(7):e0305170. doi: 10.1371/journal.pone.0305170 (PMC11271903; doi:10.1371/journal.pone.0305170)
Supplement: S3 Table — (PDF) [file pone.0305170.s003.pdf]

S3 Table Formula 1 for converting median and interquartile range (IQR) into mean and standard deviation (SD).

| Item | Formulas |
|---|---|
| Median | Median = Mean |
| IQR | IQR = 1.35 SD |
