# Supplementary material for: Predictive value of bile acids as metabolite biomarkers for gallstone disease: A systematic review and meta-analysis
Source: PLoS One. 2024 Jul 25;19(7):e0305170. doi: 10.1371/journal.pone.0305170 (PMC11271903; doi:10.1371/journal.pone.0305170)
Supplement: S4 Table — (PDF) [file pone.0305170.s004.pdf]

S4 Table The characteristics for each bile acids which performed the meta-analysis

Bile acids	Study	Data soure	Original Unit	If skewed [a]	Sample	Metabolomics
CA	Suli Jin-2022	Digit	nmol/L	1	Serum	UPLC-MS/MS
CA	Yinhuan Duan-2020	Digit	nmol/L	1	Serum	UPLC-MS/MS
CA	Han Tian-Quan-1998	Digit	µmol/L	0	Serum	GC [b]
CA	Xueping Ma-1995	Digit	µmol/L	0	Serum	HPLC [c]
CDCA	Yinhuan Duan-2020	Digit	nmol/L	1	Serum	UPLC-MS/MS
CDCA	Suli Jin-2022	Digit	nmol/L	0'	Serum	UPLC-MS/MS
CDCA	Han Tian-Quan-1998	Digit	µmol/L	0	Serum	GC [b]
CDCA	Xueping Ma-1995	Digit	µmol/L	0	Serum	HPLC [c]
DCA	Yinhuan Duan-2020	Digit	nmol/L	1	Serum	UPLC-MS/MS
DCA	Suli Jin-2022	Digit	nmol/L	1	Serum	UPLC-MS/MS
DCA	Han Tian-Quan-1998	Digit	µmol/L	0	Serum	GC [b]
DCA	Xueping Ma-1995	Digit	µmol/L	0	Serum	HPLC [c]
GCA	Zhibo Wang-2020	Digit	µmol/L	0	Serum	HPLC-MS
GCA	Suli Jin-2022	Digit	nmol/L	1	Serum	UPLC-MS/MS
GCA	Yinhuan Duan-2020	Digit	nmol/L	1	Serum	UPLC-MS/MS
GCA	Xueping Ma-1995	Digit	µmol/L	0	Serum	HPLC [c]
GCA	Chunhua Zong-1999	Digit	µmol/L	0	Serum	RP-HPLC
GCA	Dayi Chen-2001	Digit	µmol/L	0	Serum	RP-HPLC
GCDCA	Suli Jin-2022	Digit	nmol/L	1	Serum	UPLC-MS/MS
GCDCA	Yinhuan Duan-2020	Digit	nmol/L	1	Serum	UPLC-MS/MS
GCDCA	Xueping Ma-1995	Digit	µmol/L	0	Serum	HPLC [c]
GCDCA	Chunhua Zong-1999	Digit	µmol/L	0	Serum	RP-HPLC
GDCA	Zhibo Wang-2020	Digit	µmol/L	0	Serum	HPLC-MS
GDCA	Suli Jin-2022	Digit	nmol/L	1	Serum	UPLC-MS/MS

GDCA	Yinhuan Duan-2020	Digit	nmol/L	1	Serum	UPLC-MS/MS
GDCA	Xueping Ma-1995	Digit	μmol/L	0	Serum	HPLC [c]
GDCA	Chunhua Zong-1999	Digit	μmol/L	0	Serum	RP-HPLC
GDCA	Dayi Chen-2001	Digit	μmol/L	0	Serum	RP-HPLC
GUDCA	Yinhuan Duan-2020	Digit	nmol/L	1	Serum	UPLC-MS/MS
GUDCA	Suli Jin-2022	Digit	nmol/L	1	Serum	UPLC-MS/MS
LCA	Suli Jin-2022	Digit	nmol/L	1	Serum	UPLC-MS/MS
LCA	Yinhuan Duan-2020	Digit	nmol/L	1	Serum	UPLC-MS/MS
TBA	Han Tian-Quan-1998	Digit	μmol/L	0	Serum	GC [b]
TBA	Xueping Ma-1995	Digit	μmol/L	0	Serum	HPLC [c]
TBA	Chunhua Zong-1999	Digit	μmol/L	0	Serum	RP-HPLC
TCA	Zhibo Wang-2020	Digit	μmol/L	0	Serum	HPLC-MS
TCA	Yinhuan Duan-2020	Digit	nmol/L	1	Serum	UPLC-MS/MS
TCA	Suli Jin-2022	Digit	nmol/L	1	Serum	UPLC-MS/MS
TCA	Xueping Ma-1995	Digit	μmol/L	0	Serum	HPLC [c]
TCA	Chunhua Zong-1999	Digit	μmol/L	0	Serum	RP-HPLC
TCA	Dayi Chen-2001	Digit	μmol/L	0	Serum	RP-HPLC
TCDCA	Suli Jin-2022	Digit	nmol/L	1	Serum	UPLC-MS/MS
TCDCA	Yinhuan Duan-2020	Digit	nmol/L	1	Serum	UPLC-MS/MS
TCDCA	Xueping Ma-1995	Digit	μmol/L	0	Serum	HPLC [c]
TCDCA	Chunhua Zong-1999	Digit	μmol/L	0	Serum	RP-HPLC
TDCA	Yinhuan Duan-2020	Digit	nmol/L	1	Serum	UPLC-MS/MS
TDCA	Suli Jin-2022	Digit	nmol/L	1	Serum	UPLC-MS/MS
TDCA	Chunhua Zong-1999	Digit	μmol/L	0	Serum	RP-HPLC
TLCA	Zhibo Wang-2020	Digit	μmol/L	0	Serum	HPLC-MS
TLCA	Yinhuan Duan-2020	Digit	nmol/L	1	Serum	UPLC-MS/MS

TLCA	Chunhua Zong-1999	Digit	μmol/L	0	Serum	RP-HPLC
TLCA	Dayi Chen-2001	Digit	μmol/L	0	Serum	RP-HPLC
TUDCA	Yinhuan Duan-2020	Digit	nmol/L	1	Serum	UPLC-MS/MS
TUDCA	Suli Jin-2022	Digit	nmol/L	1	Serum	UPLC-MS/MS
TUDCA	Xueping Ma-1995	Digit	μmol/L	0	Serum	HPLC [c]
UDCA	Suli Jin-2022	Digit	nmol/L	1	Serum	UPLC-MS/MS
UDCA	Yinhuan Duan-2020	Digit	nmol/L	1	Serum	UPLC-MS/MS
CA	Xia Xu-2011	Digit	μg/ml	0	Bile	UPLC-MS
CA	Jingli Cai-2020	Box plot	mg/ml	1	Bile	LC-MS
CA	Yuan Liao-2021	Box plot	ng/ml	1	Bile	UPLC-ESI-MS/MS
CA	Stanislav Rejchrt-2019	Box plot	mmol/L	0	Bile	UPLC-MS
CA/CDCA	Jinpeng Chen-2003	Digit	-	0	Bile	HPLC
CA/CDCA	Saixiong Tong-1992	Digit	-	0	Bile	RP-HPLC
GCA	Yuan Liao-2021	Box plot	ng/ml	1	Bile	UPLC-ESI-MS/MS
GCA	Shaogao Liu-1990	Digit	g/L	0	Bile	HPLC
GCA	Zhiyong Dai-1997	Digit	mmol/L	0	Bile	HPLC
GCA	Jinpeng Chen-2003	Digit	mmol/L	0	Bile	HPLC
GCA	Saixiong Tong-1992	Digit	mmol/L	0	Bile	RP-HPLC
GCDCA	Yuan Liao-2021	Box plot	ng/ml	1	Bile	UPLC-ESI-MS/MS
GCDCA	Zhiyong Dai-1997	Digit	mmol/L	0	Bile	HPLC
GCDCA	Jinpeng Chen-2003	Digit	mmol/L	0	Bile	HPLC
GCDCA	Saixiong Tong-1992	Digit	mmol/L	0	Bile	RP-HPLC
GDCA	Yuan Liao-2021	Box plot	ng/ml	1	Bile	UPLC-ESI-MS/MS
GDCA	Shaogao Liu-1990	Digit	g/L	0	Bile	HPLC
GDCA	Zhiyong Dai-1997	Digit	mmol/L	0	Bile	HPLC
GDCA	Jinpeng Chen-2003	Digit	mmol/L	0	Bile	HPLC

GDCA	Saixiong Tong-1992	Digit	mmol/L	0	Bile	RP-HPLC
GLCA	Yuan Liao-2021	Box plot	ng/ml	1	Bile	UPLC-ESI-MS/MS
GLCA	Saixiong Tong-1992	Digit	mmol/L	0	Bile	RP-HPLC
TBA	M.M. Fisher (men)-1973	#N/A	mmol/L	0	Bile	GC
TBA	M.M. Fisher (women)-1973	#N/A	mmol/L	0	Bile	GC
TBA	Jinpeng Chen-2003	Digit	mmol/L	0	Bile	HPLC
TBA	Saixiong Tong-1992	Digit	mmol/L	0	Bile	RP-HPLC
TCA	Yuan Liao-2021	Box plot	ng/ml	1	Bile	UPLC-ESI-MS/MS
TCA	Shaogao Liu-1990	Digit	g/L	0	Bile	HPLC
TCA	Zhiyong Dai-1997	Digit	mmol/L	0	Bile	HPLC
TCA	Jinpeng Chen-2003	Digit	mmol/L	0	Bile	HPLC
TCA	Saixiong Tong-1992	Digit	mmol/L	0	Bile	RP-HPLC
TCDCA	Yuan Liao-2021	Box plot	ng/ml	1	Bile	UPLC-ESI-MS/MS
TCDCA	Zhiyong Dai-1997	Digit	mmol/L	0	Bile	HPLC
TCDCA	Jinpeng Chen-2003	Digit	mmol/L	0	Bile	HPLC
TCDCA	Saixiong Tong-1992	Digit	mmol/L	0	Bile	RP-HPLC
TDCA	Jingli Cai-2020	Box plot	mg/ml	1	Bile	LC-MS
TDCA	Yuan Liao-2021	Box plot	ng/ml	1	Bile	UPLC-ESI-MS/MS
TDCA	Shaogao Liu-1990	Digit	g/L	0	Bile	HPLC
TDCA	Zhiyong Dai-1997	Digit	mmol/L	0	Bile	HPLC
TDCA	Jinpeng Chen-2003	Digit	mmol/L	0	Bile	HPLC
TDCA	Saixiong Tong-1992	Digit	mmol/L	0	Bile	RP-HPLC
TLCA	Jingli Cai-2020	Box plot	mg/ml	1	Bile	LC-MS
TLCA	Saixiong Tong-1992	Digit	mmol/L	0	Bile	RP-HPLC

Note: *a* 1 means skewed; 0' means not skewed; 0 means the data repoted by mean and standard deviation. *b* GC equipped with electron capture detector. *c* HPLC and fluorescence detector.

Abbreviation: CA, Cholic Acid; CDCA, Chenodeoxycholic acid; DCA, Deoxycholic acid; GCA, Glycocholic acid; GCDCA, Glycochenodeoxycholic acid; GDCA, Glycodeoxycholic acid; GLCA, Glycolithocholic acid; GUDCA, Glycoursodeoxycholic acid; LCA, Lithocholic acid; TBA, Total bile acids; TCA, Taurocholic acid; TCDCA, Taurochenodeoxycholic acid; TDCA, Taurodeoxycholic acid; TLCA, Taurolithocholic acid; TUDCA, Tauroursodeoxycholic acid; UDCA, Ursodeoxycholic acid; 1H-NMR, Nuclear magnetic resonance; GC, Gas chromatography; GC-MS, Gas chromatography-mass spectrometry; HPLC, high performance liquid chromatography; HPLC/MS/MS, high performance liquid chromatography coupled with tandem mass spectrometry; HPLC-MS, high performance liquid chromatography-mass spectrometry; LC-MS, Liquid chromatography-mass spectrometry; RP-HPLC, Reversed phase-high performance liquid chromatography; RP-HPLC-UV, Reversed phase-high performance liquid chromatography-ultraviolet detection; UPLC-ESI-MS/MS, Ultra performance liquid chromatography-electrospray ionization-mass spectrometry; UPLC-MS, Ultra performance liquid chromatography-mass spectrometry; UPLC-MS/MS, Ultra performance liquid chromatography-mass spectrometry/mass spectrometry; UPLC-Q-TOF-MS, Ultra high performance liquid chromatography-quadrupole time-of-flight mass spectrometry.
