# Supplementary material for: Predictive value of bile acids as metabolite biomarkers for gallstone disease: A systematic review and meta-analysis
Source: PLoS One. 2024 Jul 25;19(7):e0305170. doi: 10.1371/journal.pone.0305170 (PMC11271903; doi:10.1371/journal.pone.0305170)
Supplement: S5 Table — (PDF) [file pone.0305170.s005.pdf]

S5 Table The detail of quality assessment and NOS score.

Study	Selection				Comparability	Exposure			Scores
	1) Is the case definition adequate?	2) Representa-tiveness of the cases	3) Selection of Controls	4) Definition of Controls	1) Comparability of cases and controls on the basis of the design or analysis	1) Ascertainment of exposure	2) Same method of ascertainment for cases and controls	3) Non-Response rate	
2022 Suli Jin	A *	B	A *	A *	A *	A *	A *	A *	7
2022 Man Yang	A *	B	B	A *	-	A *	A *	A *	5
2020 Linshi Wu	A *	B	C	B	A B **	A *	A *	A *	6
2020 Yinhuan Duan	A *	B	A *	A *	-	A *	A *	A *	6
2020 Zhibo Wang	A *	B	C	A *	A *	A *	A *	A *	6
2018 Zexu Chen	A *	B	A *	A *	A B **	A *	A *	A *	8
2014 DongQing Ge	A *	B	B	B	A *	A *	A *	A *	5
2001 Dayi Chen	A *	B	A *	A *	-	A *	A *	A *	6
2000 Linzi A. Thomas	A *	B	C	B	A B **	A *	A *	A *	6
1999 Chunhua Zong	A *	B	C	A *	-	A *	A *	A *	5
1998 Han Tian-Quan	A *	B	C	A *	-	A *	A *	A *	5
1995 Xueping Ma	A *	B	C	A *	-	A *	A *	A *	5
2021 Yuan Liao	A *	B	B	B	A B **	A *	A *	A *	6
2020 Jingli Cai	A *	B	B	B	A B **	A *	A *	A *	6
2019 Stanislav Rejchrt	A *	B	B	B	-	A *	A *	A *	4
2019 Natalia Molinero	A *	B	B	B	A B **	A *	A *	A *	6
2018 Wenjie Ma	A *	B	B	B	A B **	A *	A *	A *	6
2016 Wenjane Wang	A *	B	B	B	A *	A *	A *	A *	5
2011 Xia Xu	A *	B	B	B	A *	A *	A *	A *	5

2005 Bin Miao	A *	B	B	B	A *	A *	A *	A *	5
2003 Jinpeng Chen	A *	B	B	B	-	A *	A *	A *	4
2001 M Fracchia	A *	B	C	A *	-	A *	A *	A *	5
1997 Zhiyong Dai	C	B	C	B	-	A *	A *	A *	3
1993 David W. Hay	C	B	B	B	-	A *	A *	A *	3
1992 SaixiongTong	A *	B	B	B	-	A *	A *	A *	4
1990 Shaogao Liu	C	B	B	B	-	A *	A *	A *	3
1973 M.M. Fisher	A *	B	B	B	-	A *	A *	A *	4
2022 Zhiyuan Hao	A *	A *	A *	A *	A B **	A *	A *	A *	9
2015 Nirit Keren	A *	B	C	C	A *	A *	A *	A *	5
1999 Arnaldo Mamianetti	A *	B	C	B	-	A *	A *	A *	4

Note: Comparability a) study controls for age and sex; b) study controls for any additional factor:

Reference	Additional factor
2022 Zhiyuan Hao	BMI; WBC; HB; PLT; CRP; TBIL; DBIL; IBIL; ALB; GLB; ALT; AST; GGT; LDH; AKP; CRE; UA.
2021 Yuan Liao	AST; ALT; ALP; TBA; TBIL; TC; GLU; TG; HDL; LDL.
2020 Linshi Wu	BMI; AST; ALT; INR; TBIL.
2020 Jingli Cai	TG; TC; HDL; LDL; Apo A; Apo B.
2019 Natalia Molinero	HDL; Urea; CRE; AST; ALT; GGT; ALP.
2018 Zexu Chen	BMI; FGLU; TC; TG; HDL; LDL; Creatinine; BUA; AST; ALT; AFP; CEA.
2018 Wenjie Ma	TBIL; DBIL; IBIL; AST; ALT.
2000 Linzi A. Thomas	Body weight or BMI.

Abbreviation: AFP, Alpha-fetoprotein; AKP, Alkaline phosphatase; ALB, Albumin; ALP, Alkaline phosphatase; ALT, Alanine aminotransferase; Apo A, Apolipoprotein A; Apo B, Apolipoprotein B; AST, Aspartate aminotransferase; BMI, Body mass index; BUA, Blood uric acid; CEA, Carcinoembryonic antigen; CRE, Creatinine; CRP, C-reactive protein; DBIL, Direct bilirubin; FGLU, Fasting blood glucose; GGT, Glutamyl transferase; GLB, Globulin; GLU, Glucose; HB, Hemoglobin; HDL, High density lipoprotein; IBIL, Indirect bilirubin; INR, International normalized ratio; LDH, lactate dehydrogenase; LDL, Low density cholesterol; PLT, Platelet; TBA, Total bile acid; TBIL, Total bilirubin; TC, Total cholesterol; TG, Triglyceride; UA, Uric acid; WBC, White blood cell.
