# Supplementary material for: Predictive value of bile acids as metabolite biomarkers for gallstone disease: A systematic review and meta-analysis
Source: PLoS One. 2024 Jul 25;19(7):e0305170. doi: 10.1371/journal.pone.0305170 (PMC11271903; doi:10.1371/journal.pone.0305170)
Supplement: S6 Table — (PDF) [file pone.0305170.s006.pdf]

S6 Table The frequency of significant changed bile acids between case group and control group in different biological samples.

Bile acids (HMDB ID or ChEBI ID)	Blood		Bile		Stool	
	Increased	Declining	Increased	Declining	Increased	Declining
TCA (HMDB0042048)	5	2	3	1	0	0
GCDCA (HMDB0000637)	4	0	6	0	0	0
GCA (HMDB0000138)	5	1	0	4	0	0
TCDCA (CHEBI:16525)	5	1	2	1	0	0
GDCA (CHEBI:27471)	2	1	3	1	1	0
DCA (HMDB0000626)	3	1	3	0	0	1
TBA	1	0	1	2	2	0
TDCA (HMDB0000896)	2	1	3	1	0	0
UDCA (HMDB0000946)	0	3	0	0	0	1
CA (HMDB0000619)	1	1	1	2	0	0
CDCA (HMDB0000518)	1	0	1	1	0	1
LCA (HMDB0000761)	1	0	2	0	1	0
TLCA (CHEBI:36259)	1	1	1	0	0	0
TUDCA (HMDB0000874)	2	1	0	0	0	0
GUDCA (HMDB0000708)	2	0	0	0	0	0
HCA (HMDB0000760)	1	0	0	0	0	0
HDCA (HMDB0000733)	1	0	0	0	0	0
7-KetoDCA (HMDB0000391)	1	0	0	0	0	0
Total CA	0	0	0	1	0	0
Total DCA	0	0	1	0	0	0
Conjugate DCA	1	0	0	0	0	0
Unconjugated DCA	1	0	0	0	0	0
CA/CDCA	0	0	1	0	0	0

(GCA+TCA)/(GCDCA+TCDCA)	1	0	0	0	0	0
(GCA + GCDCA + GDCA)/(TCA + TDCA + TCDCA)	0	0	0	1	0	0
Conjugated/unconjugated CA	1	0	0	0	0	0
Conjugated/unconjugated CDCA	1	0	0	0	0	0
Secondary BAs	0	0	0	0	1	0
Secondary conjugate BAs	0	0	0	0	1	0
Secondary unconjugate BAs	0	0	0	0	1	0
Conjugate BAs	0	0	0	0	1	0
Conjugate primary BAs	0	1	0	0	0	0
Unconjugated BA	0	0	0	0	1	0
Glycine-conjugated BAs	0	0	0	1	0	0
Tauro-conjugated BAs	0	0	0	1	0	0
Conjugated/unconjugated primary BAs	1	0	0	0	0	0
Secondary/primary BA	1	0	0	0	0	0

Abbreviation: TCA, Taurocholic Acid; GCDCA, Glycochenodeoxycholic Acid; GCA, Glycocholic Acid; TCDCA, Taurochenodeoxycholic Acid; GDCA, lycodeoxycholic Acid; DCA, Deoxycholic Acid; TBA, Total Bile Acid; TDCA, Taurodeoxycholic Acid; UDCA, Ursodeoxycholic Acid; CA, Cholic Acid; CDCA, Chenodeoxycholic Acid; LCA, Lithocholic Acid; TLCA, Taurolithocholic Acid; TUDCA, Tauroursodeoxycholic Acid; GUDCA, Glycoursodeoxycholic Acid; HCA, Hyocholic Acid; HDCA, Hyodeoxycholic Acid; 7-KetoDCA, 7-Ketodeoxycholic Acid.
